# The mitochondrial genomes of palaeopteran insects and insights into the early insect relationships

**DOI:** 10.1038/s41598-019-54391-9

**Published:** 2019-11-28

**Authors:** Nan Song, Xinxin Li, Xinming Yin, Xinghao Li, Jian Yin, Pengliang Pan

**Affiliations:** 1 College of Plant Protection, Henan Agricultural University, Zhengzhou, 450002 China; 2 Xinyang Agriculture and Forestry University, Xinyang, 464001 China

**Keywords:** Phylogenetics, Taxonomy

## Abstract

Phylogenetic relationships of basal insects remain a matter of discussion. In particular, the relationships among Ephemeroptera, Odonata and Neoptera are the focus of debate. In this study, we used a next-generation sequencing approach to reconstruct new mitochondrial genomes (mitogenomes) from 18 species of basal insects, including six representatives of Ephemeroptera and 11 of Odonata, plus one species belonging to Zygentoma. We then compared the structures of the newly sequenced mitogenomes. A tRNA gene cluster of IMQM was found in three ephemeropteran species, which may serve as a potential synapomorphy for the family Heptageniidae. Combined with published insect mitogenome sequences, we constructed a data matrix with all 37 mitochondrial genes of 85 taxa, which had a sampling concentrating on the palaeopteran lineages. Phylogenetic analyses were performed based on various data coding schemes, using maximum likelihood and Bayesian inferences under different models of sequence evolution. Our results generally recovered Zygentoma as a monophyletic group, which formed a sister group to Pterygota. This confirmed the relatively primitive position of Zygentoma to Ephemeroptera, Odonata and Neoptera. Analyses using site-heterogeneous CAT-GTR model strongly supported the Palaeoptera clade, with the monophyletic Ephemeroptera being sister to the monophyletic Odonata. In addition, a sister group relationship between Palaeoptera and Neoptera was supported by the current mitogenomic data.

## Introduction

The acquisition of wings and of ability of flight contribute to the success of insects in the planet. The origin of insect wings and flight remain contentious. Yet, it is generally agreed that wingless species comprising the subclass Apterygota, including bristletails (Archaeognatha) and silverfish (Zygentoma), constitute the most primitive lineages in Insecta. Winged insects, namely the subclass Pterygota occur in the next stage. According to the character of whether insects can fold back the wings over the abdomen, the Pterygota are subdivided into Palaeoptera and Neoptera. The Palaeoptera includes two extant orders: Odonata (dragonflies and damselflies) and Ephemeroptera (mayflies). All other winged insects form the Neoptera. Although the monophyly of the Pterygota is well established, the interrelationships among basal orders are ambiguous. Determining the relationship of Apterygota with Pterygota and the relationship of palaeopteran orders with regard to Neoptera are the keys to understanding evolution of flight and insect diversification.

Members of two wingless insect orders Archaognatha and Zygentoma are traditionally classified in the order “Thysanura”^1,2^, based on the external morphological similarities. However, the Zygentoma was found to have a closer relationship with winged insects than has the Archaeognatha^3–8^. The mouthpart morphology supports the Dicondylia hypothesis, namely the clade Zygentoma + Pterygota. Probable synapomorphies of the Zygentoma and Pterygota were summarized by Kristensen^1^. Recent molecular studies also supported Zygentoma as a sister group to Pterygota^9,10^. The monophyly of Zygentoma was questioned because of the outside position of the *Tricholepidion*^5,11^. The relic silverfish *Tricholepidion gertschi* is the single extant species of the family Lepidotrichidae. Some morphological^5^ and molecular^11^ studies placed this species as the sister group to all other Dicondylia, while others supported a monophyletic Zygentoma^9,12–14^.

The issue on the palaeopteran relationships remains one of the major open questions in the field of insect evolution and systematics. Odonata and Ephemeroptera are the earliest-diverging lineages of winged insects. The species belonging to both orders are unable to fold their wings horizontally over their abdomen as most of neopteran insects. This character has been suggested as an evidence for the Palaeoptera monophyly. Hennig (1969)^3^ proposed some possible morphological synapomorphies of Ephemeroptera and Odonata. However, most of traits described by Hennig (1969)^3^ were considered as plesiomorphic or convergent^1,15^. A recent morphological study based on the head characters supported a monophyletic Palaeoptera^16^. In addition, a molecular study using genome-scale data^10^ supported Palaeoptera (Ephemeroptera + Odonata) and a sister group relationship of Palaeoptera to Neoptera, though with limited taxonomic sampling for palaeopteran insects.

Besides the Palaeoptera hypothesis, two other alternative hypotheses has been proposed by various authors. The hypothesis of a sister-group relationship between Odonata and Neoptera (the Metapterygota hypothesis or the basal Ephemeroptera hypothesis) was proposed based on the morphology of the wing veins, the mandibles and the respiratory system^4,17–21^. Several studies using combined molecular and morphological data also supported the basal Ephemeroptera hypothesis^6,22^. In contrast, a sister group relationship between the Odonata and the Ephemeroptera + Neoptera (the Chiastomyaria hypothesis or the basal Odonata hypothesis) was preferred by Boudreaux^5^, based primarily on the character of direct sperm transfer. Kristensen (1981)^1^ criticized Boudreaux’s characters as homoplastic. Nevertheless, several analyses based on the single molecular markers supported the basal Odonata hypothesis^12,23–25^. In addition, an analysis using data from expressed sequence tag^26^ recovered the relationship of (Odonata + (Ephemeroptera + Neoptera)), though including a single representative from each of Ephemeroptera and Odonata.

The rapid technological advance in molecular sequencing has led to acquisition of large amounts of sequence data in a very cost-effective way. At the same time, the development of assembly algorithms allows for rapidly reconstructing organelle genomes from next-generation sequencing data. Mitochondrial genomes (mitogenomes) as a class of organelle genome data are more easily to be assembled, annotated and to be more suitable for much larger-scale sampling, compared with the whole genome data. Mitochondrial phylogenomic analyses have been used to estimate the phylogenies of basal insects^27–36^. As of January 2019, there are only three mitogenome sequences available for Zygentoma in GenBank, 20 for Ephemeroptera and 27 for Odonata hinting at the need for further exploration of the mitogenomic approach in the basal insect groups.

In the present study, we sequenced a nearly complete mitogenome of *Thermobia* (Zygentoma: Lepismatidae), six partial or nearly complete mitogenomes of Ephemeroptera and 11 of Odonata, to add evidence to the controversy. Combined with published mitogenome sequences, we investigated the phylogenetic relationships of basal insects, with particular emphasis on the Palaeoptera problem.

## Materials and Methods

### Ethics statement

No specific permits were required for the insect specimen collection in this study. The specimens were collected in Santan National Forest Park, Guangshui, Hubei province, China (31.86 °N, 113.94 °E). For each species newly sequenced, 1–2 adult individuals were collected. All samples were stored in 95–100% ethanol. Voucher specimens and specimen parts after DNA extraction have been deposited at −20 °C in Entomological Museum of Henan Agricultural University.

The field studies did not involve endangered or protected species. All sequenced insects are common species in China, and are not included in the “List of Protected Animals in China”.

### DNA extraction

Total genomic DNA was extracted from the thoracic muscle tissue of each individual sample, with the TIANamp Genomic DNA Kit (TIANGEN BIOTECH CO., LTD) following the manufacturer’s protocol. Purified DNA was eluted in a single step in 50 μl Buffer TE. The concentration of extracted genomic DNA was measured by Nucleic acid protein analyzer (QUAWELL TECHNOLOGY INC.), and the average values for each species determined were shown in Table S1.

### Library construction and high throughput sequencing

Genomic DNA for each sample was pooled into twelve different libraries, respectively. Approximately equimolar amounts of genomic DNA for other insect species (*ca*. 20 different species) were mixed into the library. Each pool was designed to include distant taxonomic species in order to reduce the risk of a “contamination” and/or reads assignment errors in the following steps. For library preparation, Illumina TruSeqTM DNA Sample Prep Kit (Illumina, San Diego, CA, USA) was employed, with an average insert size of 350 bp. The indexed libraries were directly sequenced on a HiSeq X Ten platform (Beijing Novogene Bioinformatics Technology Co., Ltd, China), with 150 bp pair-ended reads.

### Reads filtering and de novo assembly

Raw reads were filtered using NGS QC Toolkit with default settings^37^. The reads containing adapters and poly-N, and low quality reads were removed. At the same time, Q20, Q30, GC-content and sequence duplication level of the cleaned data were calculated. All subsequent genome assembly were based on clean data with high quality (avg. Q20 > 90%, and avg. Q30 > 80%). *De novo* assembly for the high-quality clean reads were performed using IDBA-UD v. 1.1.1^38^. The assemblies were constructed using 200 for the setting of minimum size of contig, and an initial k-mer size of 40, an iteration size of 10, and a maximum k-mer size of 90.

### Mitogenome reconstruction and annotation

Mitogenome reconstruction method mostly followed a bioinformatics pipeline of Gillett *et al*.^39^. The mitochondrial baiting sequences (i.e., *cox1*, *cob* and *12 S*) were amplified and pre-sequenced by the primers designed by Song *et al*.^40^. The mitochondrial scaffolds were identified by blasting the mitochondrial baitings against a local database constructed by BioEdit^41^. The initial mitogenome annotations were conducted using the MITOS^42^, under default settings and the invertebrate genetic code for mitochondria. The gene boundaries were further checked and refined by alignment with homologous sequences of related species (see details in Table S1) in MEGA 7^43^. Mappings to the mitochondrial contigs were performed using BWA v. 0.7.5^44^. Alignments produced in SAM format were converted to sorted BAM format by SAMtools v. 0.1.19^45^. Statistics for nucleotide coverage were generated with Qualimap v.2.2.1^46^.

Representative specimens were identified to species or genus level by checking adult morphological characters, and by Blast matches to *cox1* records from the BOLD database (http://www.boldsystems.org/) and NCBI Genbank (http://www.ncbi.nlm.nih.gov/genbank/). The detailed classification information, voucher numbers of species sequenced and accession numbers of the new mitogenome sequences are given in Table S1.

### Multiple sequence alignments

Each mitochondrial protein-coding gene was aligned separately based on the corresponding amino acid translations using the MUSCLE algorithm^47^, as implemented in TranslatorX^48^. All protein-coding gene alignments were concatenated by using FASconCAT-G^49^ to construct the dataset of PCG1_NT_2_NT_3_NT_. The yn00 program of the PAML package^50^ was used to calculate the nonsynonymous (*dN*) and synonymous (*dS*) substitution rates of the concatenated 13 protein-coding genes, with the method of Yang and Nielsen^51^. DAMBE 7^52^ was used to conduct tests for substitution saturation of each codon position. According to the index of substitution saturation, the third codon positions were significantly saturated (*Iss* > *Iss.cSym* and *Iss* > *Iss.cAsym*) (Table 1). To account for the effect of substitution saturation, two approaches were employed in the further phylogenetic analyses. First, the protein-coding genes were re-concatenated with FASconCAT-G under the parameter option of 3rd sequence position exclusion in order to create a dataset with codon positions 1 and 2 (PCG1_NT_2_NT_). Second, the protein-coding genes were re-concatenated with FASconCAT-G under the parameter option of RY-coding of 3rd sequence positions to compile a dataset with the third codon position nucleotides recoded into two state categories, R (purine) and Y (pyrimidine)^53^ (PCG1_NT_2_NT_3_RY_).

**Table 1:** Saturation test based on the datasets of 85taxa_PCG1_NT_2_NT_3_RY_RNA.

Gene partitions	NumOTU	Iss	Iss.cSym	Psym	Iss.cAsym	Pasym
PCG1_NT_	32	0.516	0.808	0.000	0.554	0.000
PCG2_NT_	32	0.371	0.808	0.000	0.554	0.000
PCG3_NT_	32	0.897	0.808	0.000	0.554	0.000
rRNA	32	0.703	0.790	0.000	0.520	0.000
tRNA	32	0.689	0.793	0.000	0.524	0.000

Note: two-tailed tests are used.

Ribosomal and transfer RNA genes were aligned individually with MAFFT^54^ under the iterative refinement method incorporating the most accurate local pairwise alignment information (E-INS-i). Gaps of alignments were striped by Gap Strip/Squeeze v2.1.0 with 40% Gap tolerance (http://www.hiv.lanl.gov/content/sequence/GAPSTREEZE/gap.html). The dataset PCG1_NT_2_NT_ was concatenated with the tRNA and rRNA datasets in order to create a combined dataset of PCG1_NT_2_NT_RNA. The PCG1_NT_2_NT_3_RY_ dataset, tRNA dataset and rRNA dataset were combined together to construct the PCG1_NT_2_NT_3_RY_RNA dataset. Multiple sequence alignments were statistically scored using AliStat^10^. Nucleotide compositions of the mitogenome sequences were estimated using MEGA 7^43^.

### Phylogenetic inference

A total of eighty-five species were included to create the full taxon dataset. Of which, three species representing three families in Zygentoma, eighteen representing three families in Ephemeroptera and 31 representing 17 families in Odonata were used in the phylogenetic analysis. In addition, six species of Archaeognatha and 20 species representing 17 orders of Neoptera were also included in the analysis. Two species of Collembola and five of Diplura were selected as outgroups.

Phylogenetic trees were reconstructed using both maximum likelihood (ML) and Bayesian inferences (BI). Partitioned ML analyses were performed with the IQ-TREE^55^, as implemented in the CIPRES Science Gateway^56^. The 13 protein-coding genes were partitioned by gene, whereas the 22 tRNA genes and the two rRNA genes were considered as two separate partitions. The best partitioning schemes (Table S2) for the datasets were selected with PartitionFinder 2^57^. The site-homogeneous GTR model was often chosen as the best-fit model for each partition in every dataset (Table S2). We performed 10,000 ultrafast^58^ bootstrap replicates to investigate nodal support across the topology.

Ten-fold Bayesian cross-validation analyses were performed to test the fit of the site-heterogeneous mixture model CAT-GTR and the site-homogeneous model GTR to our full taxon data (85taxa_PCG1_NT_2_NT_3_RY_RNA and 85taxa_PCG1_NT_2_NT_RNA) using PhyloBayes 3.3f^59^. The results showed that the CAT-GTR model was the best fitting model for both datasets (Table 2). BI analyses were conducted with PhyloBayes MPI^60,61^ as implemented in the CIPRES Science Gateway^56^, under the CAT-GTR model. For each analysis, two Markov chain Monte Carlo (MCMC) chains were run in parallel, after the removal of constant sites from the alignments. The minimum number of cycles were set to 20,000. Stationarity was considered to be reached when the maxdiff was <0.3 and minimum effective size was >50. The run would be terminated if analysis passed the convergence test. The first 1000 trees of each MCMC were treated as the burn-in, and the majority-rule consensus tree was calculated from the saved trees.

**Table 2:** Cross-validation analyses of the homogeneous and heterogeneous models implemented in PhyloBayes based onnucleotide datasets.

Dataset	Reference model	Model used	Cross-validation score	Standard deviation
85taxa_PCG1_NT_2_NT_3_RY_RNA	GTR	CAT-GTR	1860.73	±106.882
85taxa_PCG1_NT_2_NT_RNA	GTR	CAT-GTR	1864.43	±163.653

Positive scores: better than reference model (GTR).

The preliminary full taxon (85 taxa) data ML trees were used for the RogueNaRok^62^ analysis, which can identified the taxa being assumed to show uncertain phylogenetic position. The result suggested *Epiophlebia superstes* (Odonata) as a rogue taxon leading to less accurate overall phylogenetic reconstructions. In addition, both ML trees displayed the obviously long branch lengths leading to the outgroup taxa from the Collembola (*Bilobella aurantiaca*, *Cryptopygus antarcticus*) and Diplura (*Lepidocampa weberi*, *Campodea fragilis*, *Campodea lubbocki*). To reduce the potential effect of problematic taxa on the recovered topology, we created the reduced taxon (79 taxa) data which excluded five outgroup taxa mentioned above and the rogue species identified by RogueNaRok. Both ML and Bayesian analyses were repeated based on the reduced taxon datasets (79taxa_PCG1_NT_2_NT_3_RY_RNA and 79taxa_PCG1_NT_2_NT_RNA), with the settings as the analyses from the full taxon data.

## Results

### Genome sequencing

Eighteen partial or nearly complete mitogenomes were newly determined for 11 species of Odonata (four dragonflies and seven damselflies), six species of Ephemeroptera and one species of Zygentoma by using a next-generation sequencing method. After filtering, the number of Illumina reads obtained varied from 57,181,127 reads to 83,346,749 reads. In most cases, 0.01% to 0.09% corresponded to mitochondrial reads. Through the BLAST-searches with baiting sequences, the mitogenome identified for each newly sequenced species was assembled on a single contig. The analyzed mitogenomes are around 15,000 nt in size, except for *Agriocnemis femina*, *Orthetrum albistylum*, *Orthetrum melania* and *Cloeon dipterum*. The four species are exhibiting a size ranging 5,540~13,603 nt. The sequencing coverage for each mitochondrial contig varied from 9-fold for *C. dipterum* to 2,685-fold for *Ephemera* sp. (Table 3).

**Table 3:** The statistics of each mitogenomic contig assembled in this study.

Order	Name	Length	Mapped bases	Mean coverage	Mapped reads
Ephemeroptera	*Cloeon dipterum*	5,540	50,549	9	337
Ephemeroptera	*Epeorus* sp.	15,355	1,599,545	104	10,664
Ephemeroptera	*Rhithrogena* sp.	15,277	2,434,743	159	16,232
Ephemeroptera	*Parafronurus* sp.	15,214	9,525,907	626	63,510
Ephemeroptera	*Isonychia* sp.	15,830	35,145,298	2,220	468,740
Ephemeroptera	*Ephemera* sp.	15,683	42,106,306	2,685	561,487
Odonata	*Orthetrum albistylum*	10,296	382,641	37	2,551
Odonata	*Orthetrum melania*	8,609	320,544	37	2,137
Odonata	*Ischnura elegans*	14,990	1,075,720	72	7,172
Odonata	*Paracercion malayanum*	15,366	815,970	53	5,446
Odonata	*Agriocnemis femina*	13,603	1,157,744	85	7,719
Odonata	*Platycnemis phyllopoda*	15,125	2,444,403	162	16,297
Odonata	*Coeliccia cyanomelas*	15,100	2,869,718	190	19,133
Odonata	*Sympetrum eroticum*	14,940	6,600,519	442	44,012
Odonata	*Mesopodagrion tibetanum*	15,170	10,327,869	681	68,857
Odonata	*Anotogaster sieboldii*	15,132	10,525,046	696	70,170
Odonata	*Mnais tenuis*	14,632	10,687,092	730	142,504
Zygentoma	*Thermobia* sp.	16,586	13,047,410	787	87,090

**Figure 1:**
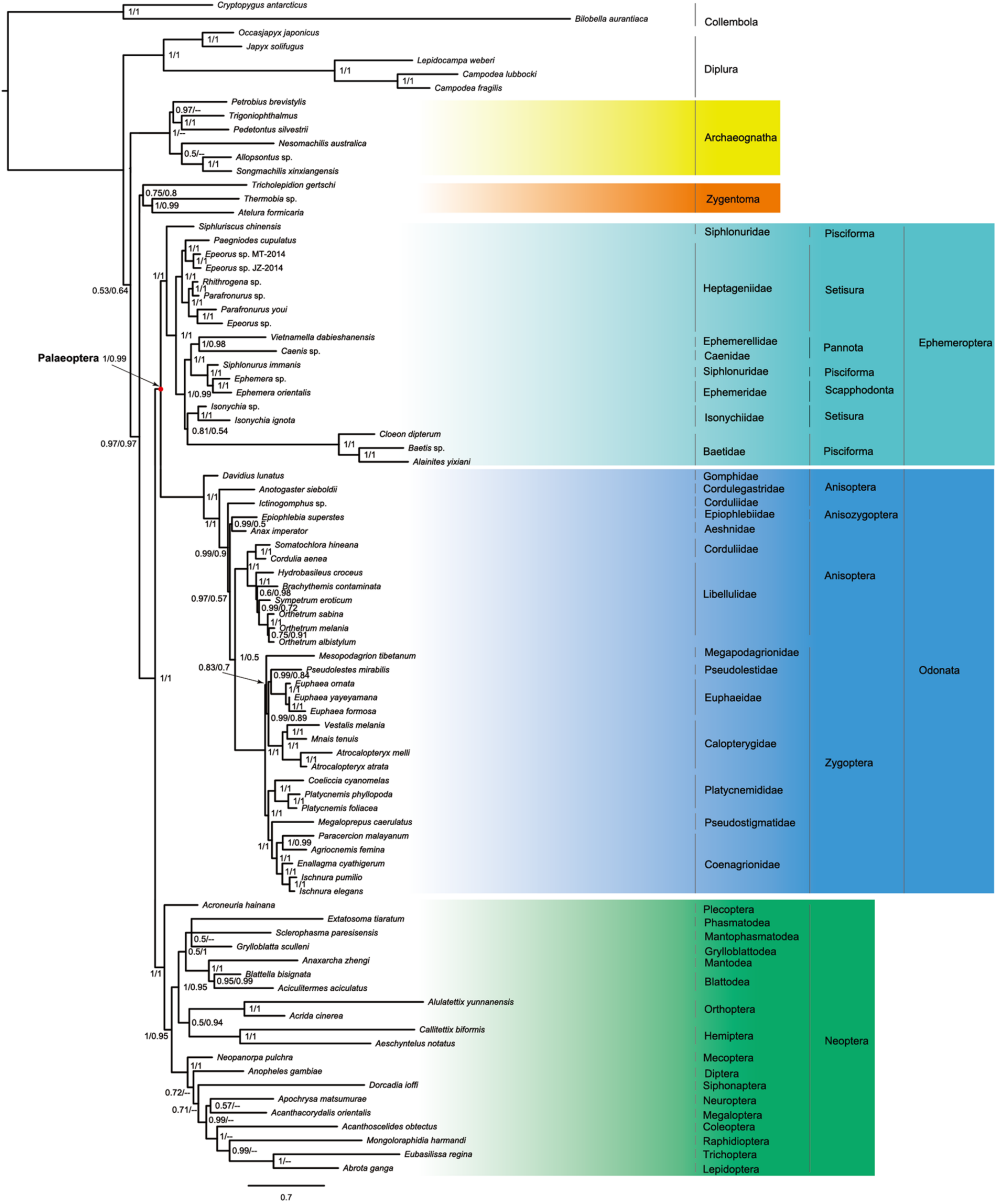
Organization of mitochondrial genomes newly sequenced in this study. The genes above the line indicate the transcriptional direction being from left to right, while those below the line indicate the transcriptional direction being from right to left.

In the fourteen nearly complete mitogenomes (14,632 nt ~ 16,586 nt), we were able to determine their gene arrangement (Fig. 1). The mitochondrial gene order was conserved inside of these newly sequenced species, with the same gene organization as the ancestral insect^63^. A total of 35~37 mitochondrial genes were identified in each nearly complete mitogenome (Fig. 1). The *trnF* was missing in the species of *Coeliccia cyanomelas*, *Parafronurus* sp. and *Rhithrogena* sp. The *trnG* is missing in *Anotogaster sieboldi* and *Sympetrum eroticum*. The *trnP* in *Sympetrum eroticum*, the *trnL(tag)* in *Mnais tenuis* and the *trnS(tga)* in *Platycnemis phyllopoda* were not detected. For the three partial mitogenome sequences with the length of 8,528 nt (*O. melania*), 10,107 nt (*O. albistylum*) and 13,280 nt (*A. femina*), the missing genes were mainly located adjacent to the putative control region. In the species of *C. dipterum*, only nineteen mitochondrial genes were detected (Fig. 1), with a total length of 5,540 nt. Failure to reconstruct the mitogenome of this species may be owing to the lower sequencing depth (Table 3). The mitogenome of the *Thermobia* sp. (Zygentoma) has the AT content of 68% (Table 4). Among the analyzed palaeopteran species, the mean AT content for the new mitogenomes of six mayflies is also 68%, which is lower than the new mitogenomes of four dragonflies (avg. 71%) and of seven damselflies (avg. 72%).

**Table 4:** Nucleotide compositions and substitution rates estimated for major lineages.

Order	avg. AT	avg. CG	*dN*	*dS*	*dN*/*dS*
Collembola	69.47	30.53	0.2572	4.7292	0.0544
Diptera	76.57	23.43	0.1685	4.5708	0.0369
Archaeognatha	69.72	30.28	0.1981	4.7253	0.0419
Zygentoma	65.47	34.53	0.1992	4.7625	0.0418
Ephemeroptera	65.67	34.33	0.1791	4.7496	0.0377
Odonata	70.85	29.15	0.1668	4.6642	0.0357
Plecoptera	61.71	38.29	0.1825	4.7947	0.0381
Phasmatodea	75.46	24.54	0.2358	4.6031	0.0512
Mantophasmatodea	74.34	25.66	0.1914	4.6266	0.0414
Grylloblattodea	70.26	29.74	0.1858	4.7594	0.0390
Mantodea	77.22	22.78	0.2028	4.5322	0.0447
Blattodea	71.01	28.99	0.1822	4.6726	0.0390
Orthoptera	74.94	25.06	0.2175	4.6027	0.0472
Hemiptera	75.69	24.31	0.2337	4.6008	0.0508
Mecoptera	76.00	24.00	0.1652	4.5584	0.0362
Diplura	67.12	32.88	0.2160	4.7141	0.0458
Siphonaptera	78.32	21.68	0.1997	4.5806	0.0436
Neuroptera	76.66	23.34	0.1786	4.5539	0.0392
Megaloptera	75.86	24.14	0.1751	4.6050	0.0380
Coleoptera	75.48	24.52	0.2099	4.6733	0.0449
Raphidioptera	78.83	21.17	0.2123	4.5821	0.0463
Trichoptera	77.80	22.20	0.2253	4.6471	0.0485
Lepidoptera	80.54	19.46	0.1999	4.5499	0.0439

### Phylogenetic analyses

The full taxon protein-coding gene dataset including all codon positions (PCG1_NT_2_NT_3_NT_) comprises 11,070 nucleotides. Completeness score (Ca) calculated by AliStat for the alignment PCG1_NT_2_NT_3_NT_ was 0.9624. The substantial missing data occurred in the species of *C. dipterum*. The alignment PCG1_NT_2_NT_3_RY_RNA comprises approximately 15,218 base positions (Ca = 0.9423). After excluding the third positions, the reduced site matrix PCG1_NT_2_NT_RNA contains 11,528 nucleotide positions (Ca = 0.9359).

The rates of nonsynonymous substitutions (*dN*) ranged from 0.1652 (Mecoptera) to 0.2572 (Collembola), and the rates of synonymous substitutions (*dS*) ranged from 4.5322 (Mantodea) to 4.7947 (Plecoptera) (Table 4). Both Odonata and Ephemeroptera had the relatively slow nonsynonymous substitution rates (Odonata: 0.1668 and Ephemeroptera: 0.1791) and the lower ratios of nonsynonymous/synonymous substitution rates (Odonata: 0.0357 and Ephemeroptera: 0.0377).

Partitioned ML analyses (IQTRE, GTR model) and Bayesian inference (PhyloBayes, CAT-GTR model) presented the conflict hypotheses. But data treatment methods (excluding or RY-coding the third positions) had no significant effect on tree reconstructions, under the same inference method.

Phylogenetic trees from ML analyses (Fig. S1) recovered Odonata as a sister group to Neoptera, but with low-to-moderate support (bootstrap support, BP ≤77). The Ephemeroptera emerged as monophyletic and as sister group to the single representative of Plecoptera (*Acroneuria hainana*). The Ephemeroptera + Plecoptera clade was the sister group to the remaining pterygote orders. Thus, ML trees substantially supported the basal Ephemeroptera hypothesis. In addition, the following higher-level relationships were consistent across the ML analyses: the monophyly of the Zygentoma was well supported (BP ≥ 98) and *T. gertschi* was retrieved as the first clade within this order. The Zygentoma was placed as sister group of all the winged insects, rendering the Apterygota paraphyletic. In the current data matrices, the taxon sampling focused on the Ephemeroptera and Odonata. Thus, the monophyly of some higher-level lineages in both orders can be tested. At the suborder level, the Setisura, Scapphodonta and Pannota were monophyletic within Ephemeroptera. However, the Pisciforma was paraphyletic with respect to *Siphluriscus chinensis*. Within Odonata, the clade Anisoptera including the single representative of Anisozygoptera (*Epiophlebia superstes*) formed a strongly supported sister group relationship to the monophyletic Zygoptera (BP = 100). The reduced taxon datasets (79taxa_PCG1_NT_2_NT_3_RY_RNA and 79taxa_PCG1_NT_2_NT_RNA) resulted in a largely identical ingroup tree topology (Fig. S2) to those inferred from the full taxon datasets.

**Figure 2:**
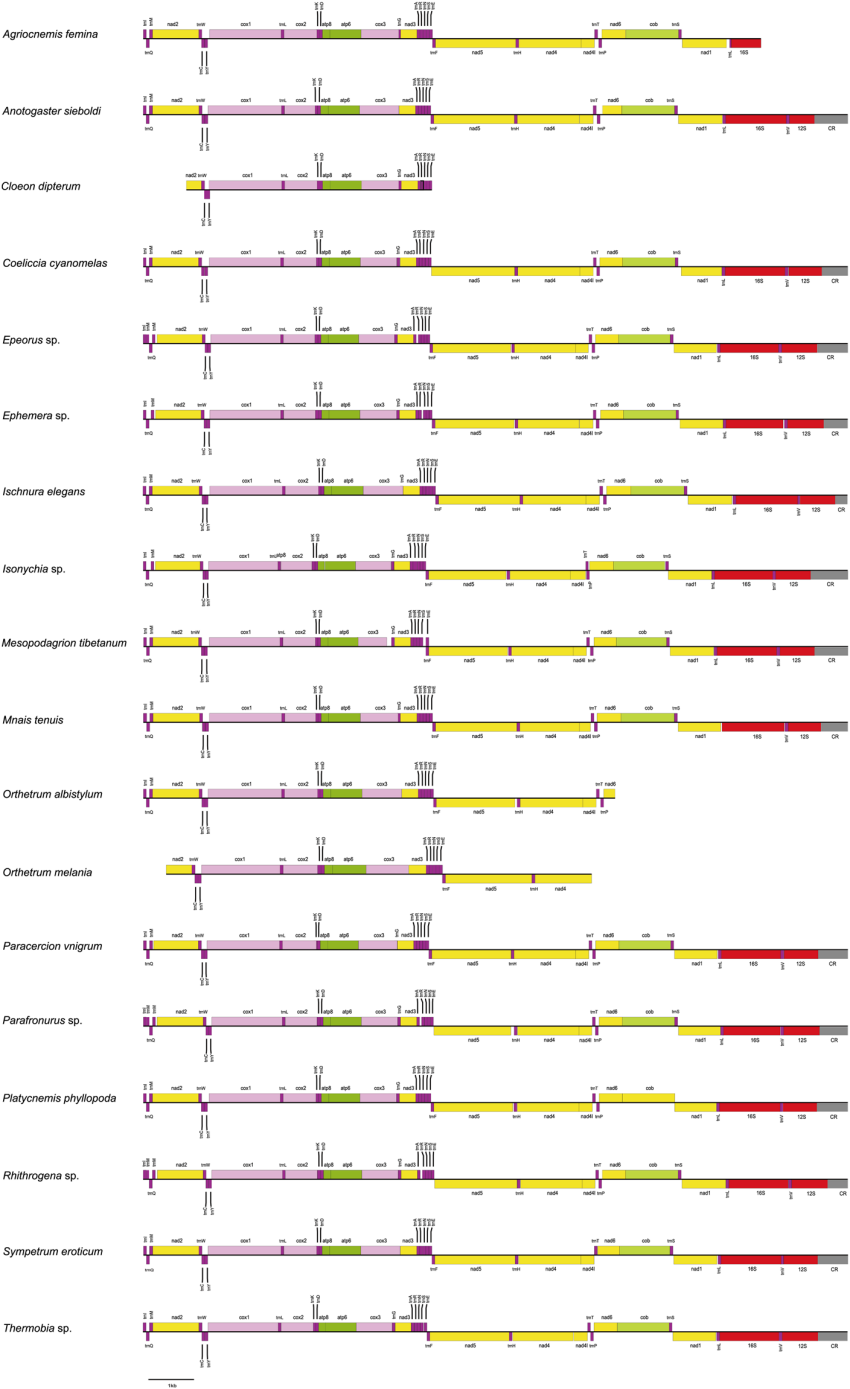
Phylogenetic tree based on Bayesian inference of the nucleotide sequence dataset of 85taxa_PCG1_NT_2_NT_3_RY_RNA, under the CAT + GTR model. Values at nodes are Bayesian posterior probability support (Left: the values from dataset of 85taxa_PCG1_NT_2_NT_3_RY_RNA, right: the values from dataset of 85taxa_PCG1_NT_2_NT_RNA). “–” indicates the node not being recovered by the dataset of 85taxa_PCG1_NT_2_NT_RNA. Scale bar represents substitutions/site. The meaning of color is as follows: yellow, Archaeognatha; orange, Archaeognatha; wathet, Ephemeroptera; blue, Odonata; green, Neoptera.

In contrast, Bayesian analyses under the CAT-GTR model consistently supported the Palaeoptera hypothesis (Fig. 2), with strong nodal support values (posterior probabilities, PP = 1 or PP = 0.99). The monophyly of Zygentoma was recovered in Bayesian analyses, but with lower support values (PP < 0.9). In addition, the subclass Dicondylia was supported because of the sister group relationship between the Zygentoma and pterygote insects (PP = 0.97). The Plecoptera formed a sister group of all other Neoptera. Therefore, the monophyly of Neoptera were supported by the Bayesian inference under the site-heterogeneous CAT-GTR model. Removal of the long-branched outgroup taxa and the rogue species had no significant influence on the ingroup topology, except for the Zygentoma. In the Bayesian analyses with the reduced taxon datasets, the Zygentoma was recovered as paraphyletic with respect to *T. gertschi* (Fig. S3).

## Discussion

### Characteristics of the new mitogenomes

The average AT content of the six newly sequenced mayflies is 68%, which is slightly higher than the average AT content of the whole Ephemeroptera (65.67%). Yet it is still the lowest proportion compared to other insect orders included (Table 4). This result is in agreement with the previous studies^29,36,64,65^. For the newly determined mayflies, there is a tRNA gene cluster of IMQM found in three species, namely, *Parafronurus* sp., *Rhithrogena* sp. and *Epeorus* sp. (Fig. 1). The three species belong to the family Heptageniidae. All previously published mitogenomes from Heptageniidae, except for *Paegniodes cupulatus*, exhibit the arrangement of IMQM^29,36,66,67^. In contrast, the mitogenomes from other families in Ephemeroptera^27,65,67–72^ have the ancestral gene order, with the typical IQM tRNA arrangement. The gene rearrangement with IMQM tRNA cluster may serve as a potential synapomorphy for the family Heptageniidae. Further studies needs expanding mitogenomic taxon sampling from Ephemeroptera to affirm this point.

### Relationships among the basal winged insects

Some previous studies have attempted to resolve the relationships among the basal winged insects and to address the Palaeoptera problem, based on the data from molecular sequences^7,8,10,12,26–28,31,36,68,70,73^ and/or morphological characters^9,16^. However, conflicting results were obtained due to limited taxon sampling and various analysis methods (Table S3).

In our analyses, some taxa exist as long branches, for example, the outgroup taxa from Collembola. The tree with long branches may be problematic for accurate estimation of the phylogenetic relationships. The long branch attraction (LBA) artefact^74–76^ is a common phenomenon occurred in the tree reconstructions, where unrelated species can be grouped together artifactually due to the shared long branch lengths. The mitogenome sequences from some insect lineages show the specific evolution rate and the distinct base composition^40,77–79^. Both factors may have a negative impact on the phylogenetic reconstruction from the mitogenomic data. To assess the effect of taxon sampling on our results, we removed the long-branched outgroups and the rogue species (namely that with unstable placement across the trees). However, the results gave rise to the virtually same topologies when analyzing the reduced sequence data under the same inference method.

Two previous study based on the complete mitogenomes^27,36^ supported the basal Ephemeroptera hypothesis. Nevertheless, other mitogenomic studies^31,70,73^ with various taxon sampling supported the basal position of Odonata. Therefore, the previous studies based on the complete mitogenomes yielded conflict results for the phylogenetic relationships among the most basal extant pterygote lineages. In this study, with the site-homogenous GTR model, all ML trees recovered a sister group relationship between Odonata and the majority of Neoptera, with weak to moderate support. An abnormal placement of Plecoptera were retained in the ML trees, where the Plecoptera was placed far away from other neopteran insects and appeared a sister group to the monophyletic Ephemeroptera. Both Plecoptera and Ephemeroptera were sister to all the remaining pterygote orders. This arrangement is similar to the basal Ephemeroptera hypothesis, and is in agreement with the studies by Zhang *et al*.^36^ and Cai *et al*.^27^.

On the current mitogenomic data, the Bayesian inference analyses with the site-heterogeneous CAT-GTR model yielded topologies consistently supporting the Palaeoptera hypothesis, and suggested that the monophyletic Ephemeroptera is sister group to the monophyletic Odonata. In addition, Bayesian inferences resulted in a monophyletic Neoptera, in which the Plecoptera was sister to all other neopteran lineages (Figs. 2 and S3). Both the prior studies^77,78^ and the cross-validation analyses conducted in this study indicated that the site-heterogeneous CAT-GTR model implemented in the PhyloBayes software is more fitting for modeling the evolution of insect mitogenomes than the site-homogenous GTR model. Moreover, the site-heterogeneous CAT-GTR model has been shown to be least sensitive to long-branch attraction phenomena^40,77–80^. From a point of view of morphology, a prior study by Blanke *et al*.^16^ have refuted the possibility of a sister relationship between Plecoptera and Ephemeroptera. All lineages with the sequences that branch near the base of the tree suffer from relatively low apparent substitution rates, which include Plecoptera and Ephemeroptera. The shared sequence similarity might contribute to a kind of convergence that could lead to an artificially deep branching position of the Plecoptera in the ML trees. Therefore, the phylogenetic results from the Bayesian inference analyses using the site-heterogeneous CAT-GTR model (Figs. 2 and S3) should be closer to the species tree.

The Palaeoptera hypothesis was first proposed by Martynow (1924)^81^ and Crampton (1924)^82^. Support for a monophyletic origin of Odonata and Ephemeroptera includes various evidence from the morphological characteristics, namely, the shortened antennae^3^, aquatic larvae^82^, the distinct wing joint^83–85^, and a paired penis^86^. Especially, the monophyly of Palaeoptera was supported by head structures in a recent morphological study of Blanke *et al*.^16^. Several molecular studies using nuclear genes supported the Palaeoptera^6,7,9,87–89^. A more recent phylogenomic study also suggested that the Ephemeroptera and Odonata derived from a common ancestor^10^. This study is the first to provide the mitogenomic data supporting a sister group relationship between the monophyletic Ephemeroptera and Odonata. In addition, a sister group relationship of Palaeoptera and Neoptera is strongly supported. The results presented here would be expected to be confirmed by further studies with more extensive taxon sampling.

## Supplementary information

Supplementary Files
