# Supplementary material for: The mitochondrial genomes of palaeopteran insects and insights into the early insect relationships
Source: Sci Rep. 2019 Nov 28;9:17765. doi: 10.1038/s41598-019-54391-9 (PMC6883079; doi:10.1038/s41598-019-54391-9)
Supplement: Supplementary file 1 — Supplementary Files [file 41598_2019_54391_MOESM1_ESM.pdf]

The mitochondrial genomes of palaeopteran insects and insights into the early insect relationships

Nan Song[1,*], Xinxin Li[1], Xinming Yin[1], Xinghao Li[1], Jian Yin[2], Pengliang Pan[2]

[1]College of Plant Protection, Henan Agricultural University, Zhengzhou 450002, China

[2]Xinyang Agriculture and Forestry University, Xinyang 464001, China

[*]Corresponding authors, e-mail: songnan@henau.edu.cn

Table S1. Taxa included in this study.

Item	Order	Suborder	Superfamily	Family	Species	Accession number	Voucher numbers for newly sequenced species	DNA conc. (ng/ul)
Outgroup	Collembola	Entomobryomorpha	Isotomoidea	Isotomidae	*Cryptopygus antarcticus*	EU016194	-	-
	Collembola	Poduromorpha	Poduroidea	Neanuridae	*Bilobella aurantiaca*	EU084034	-	-
	Diplura	Dicellurata	Japygoide	Japygidae	*Occasjapyx japonicus*	JN990600	-	-
	Diplura	Dicellurata	Japygoide	Japygidae	*Japyx solifugus*	AY771989	-	-
	Diplura	Rhabdura	Campodeoidea	Campodeidae	*Lepidocampa weberi*	JN990601	-	-
	Diplura	Rhabdura	Campodeoidea	Campodeidae	*Campodea fragilis*	DQ529236	-	-
	Diplura	Rhabdura	Campodeoidea	Campodeidae	*Campodea lubbocki*	DQ529237	-	-
	Archaeognatha	-	-	-	*Nesomachilis australica*	AY793551	-	-
	Archaeognatha	-	-	-	*Allopsontus* sp.	KJ754500	-	-
	Archaeognatha	-	-	-	*Songmachilis xinxiangensis*	JX308221	-	-
	Archaeognatha	-	-	-	*Petrobius brevistylis*	AY956355	-	-
	Archaeognatha	-	-	-	*Trigoniophthalmus*	EU016193	-	-
	Archaeognatha	-	-	-	*Pedetontus silvestrii*	EU621793	-	-
	Zygentoma	-	-	Nicoletiidae	*Atelura formicaria*	EU084035	-	-
	Zygentoma	-	-	Lepismatidae	***Thermobia* sp.**	MK951657	EMHAU_16071107	270.03
	Zygentoma	-	-	Lepidotrichidae	*Tricholepidion gertschi*	AY191994	-	-
	Ephemeroptera	Pisciforma	-	Baetidae	***Cloeon dipterum***	MK951672	EMHAU_16071111	559.50
	Ephemeroptera	Pisciforma	-	Baetidae	*Baetis* sp.	GU936204	-	-
	Ephemeroptera	Pisciforma	-	Baetidae	*Alainites yixiani*	GU479735	-	-
	Ephemeroptera	Pannota	-	Caenidae	*Caenis* sp. YJ-2009	GQ502451	-	-
	Ephemeroptera	Pannota	-	Ephemerellidae	*Vietnamella dabieshanensis*	HM067837	-	-
	Ephemeroptera	Scapphodonta	-	Ephemeridae	***Ephemera* sp.**	MK951659	EMHAU_16071112	23.73
	Ephemeroptera	Scapphodonta	-	Ephemeridae	*Ephemera orientalis*	NC_012645	-	-
	Ephemeroptera	Setisura	-	Heptageniidae	***Rhithrogena* sp.**	MK951670	EMHAU_16071115	21.57
	Ephemeroptera	Setisura	-	Heptageniidae	***Parafronurus* sp.**	MK951673	EMHAU_16082304	327.47

	Ephemeroptera	Setisura	-	Heptageniidae	***Epeorus* sp.**	MK951674	EMHAU_16082311	245.10
	Ephemeroptera	Setisura	-	Heptageniidae	*Paegniodes cupulatus*	HM004123	-	-
	Ephemeroptera	Setisura	-	Heptageniidae	*Epeorus* sp. JZ-2014	KJ493406	-	-
	Ephemeroptera	Setisura	-	Heptageniidae	*Epeorus* sp. MT-2014	KM244708	-	-
	Ephemeroptera	Setisura	-	Heptageniidae	*Parafronurus youi*	EU349015	-	-
	Ephemeroptera	Setisura	-	Isonychiidae	***Isonychia* sp.**	MK951658	EMHAU_16071109	451.00
	Ephemeroptera	Setisura	-	Isonychiidae	*Isonychia ignota*	HM143892	-	-
	Ephemeroptera	Pisciforma	-	Siphlonuridae	*Siphluriscus chinensis*	HQ875717	-	-
	Ephemeroptera	Pisciforma	-	Siphlonuridae	*Siphlonurus immanis*	FJ606783	-	-
	Odonata	Anisoptera	-	Aeshnidae	*Anax imperator*	KX161841	-	-
	Odonata	Anisoptera	-	Cordulegastridae	***Anotogaster sieboldii***	MK951663	EMHAU_16071526	247.77
	Odonata	Anisoptera	-	Corduliidae	*Somatochlora hineana*	MG594801	-	-
	Odonata	Anisoptera	-	Corduliidae	*Cordulia aenea*	JX963627	-	-
	Odonata	Anisoptera	-	Gomphidae	*Davidius lunatus*	NC_012644	-	-
	Odonata	Anisoptera	-	Gomphidae	*Ictinogomphus* sp.	KM244673	-	-
	Odonata	Anisoptera	-	Libellulidae	*Orthetrum sabina*	KU361234	-	-
	Odonata	Anisoptera	-	Libellulidae	***Orthetrum melania***	MK951662	EMHAU_16071517	28.50
	Odonata	Anisoptera	-	Libellulidae	***Orthetrum albistylum***	MK951665	EMHAU_16071520	110.80
	Odonata	Anisoptera	-	Libellulidae	***Sympetrum eroticum***	MK951664	EMHAU_16071528	187.87
	Odonata	Anisoptera	-	Libellulidae	*Brachythemis contaminata*	KM658172	-	-
Ingroup	Odonata	Anisoptera	-	Libellulidae	*Hydrobasileus croceus*	KM244659	-	-
	Odonata	Anisozygoptera	-	Epiophlebiidae	*Epiophlebia superstes*	JX050223	-	-
	Odonata	Zygoptera	-	Calopterygidae	*Vestalis melania*	JX050224	-	-
	Odonata	Zygoptera	-	Calopterygidae	***Mnais tenuis***	MK951660	EMHAU_16071524	81.73
	Odonata	Zygoptera	-	Calopterygidae	*Atrocalopteryx atrata*	NC_027181	-	-
	Odonata	Zygoptera	-	Calopterygidae	*Atrocalopteryx melli*	MG011692	-	-
	Odonata	Zygoptera	-	Coenagrionidae	***Paracercion malayanum***	MK951669	EMHAU_15122908	11.27
	Odonata	Zygoptera	-	Coenagrionidae	***Agriocnemis femina***	MK951667	EMHAU_16071508	317.33
	Odonata	Zygoptera	-	Coenagrionidae	*Enallagma cyathigerum*	MF716899	-	-
	Odonata	Zygoptera	-	Coenagrionidae	***Ischnura elegans***	MK951668	EMHAU_16071502	202.60

Odonata	Zygoptera	-	Coenagrionidae	*Ischnura pumilio*	KC878732	-	-
Odonata	Zygoptera	-	Euphaeidae	*Euphaea formosa*	HM126547	-	-
Odonata	Zygoptera	-	Euphaeidae	*Euphaea ornata*	NC_026059	-	-
Odonata	Zygoptera	-	Euphaeidae	*Euphaea yayeyamana*	KF718293	-	-
Odonata	Zygoptera	-	Megapodagrionidae	***Mesopodagrion tibetanum***	MK951671	EMHAU_16071523	249.90
Odonata	Zygoptera	-	Platycnemididae	***Coeliccia cyanomelas***	MK951666	EMHAU_16071505	1242.83
Odonata	Zygoptera	-	Platycnemididae	***Platycnemis phyllopoda***	MK951661	EMHAU_16071521	102.23
Odonata	Zygoptera	-	Platycnemididae	*Platycnemis foliacea*	KP233804	-	-
Odonata	Zygoptera	-	Pseudolestidae	*Pseudolestes mirabilis*	FJ606784	-	-
Odonata	Zygoptera	-	Pseudostigmatidae	*Megaloprepus caerulatus*	KU958377	-	-
Plecoptera	-	Perloidea	Perlidae	*Acroneuria hainana*	KM199685	-	-
Mecoptera	-	-	Panorpidae	*Neopanorpa pulchra*	FJ169955	-	-
Diptera	Nematocera	Culicoidea	Culicidae	*Anopheles gambiae*	MG753657	-	-
Neuroptera	Hemerobiiformia	-	Chrysopidae	*Apochrysa matsumurae*	AP011624	-	-
Megaloptera	-	-	Corydalidae	*Acanthacorydalis orientalis*	KF840564	-	-
Siphonaptera	-	-	Vermipsyllidae	*Dorcadia ioffi*	MF124314	-	-
Coleoptera	Polyphaga	Chrysomeloidea	Chrysomelidae	*Acanthoscelides obtectus*	KX825864	-	-
Raphidioptera	-		Raphidiidae	*Mongoloraphidia harmandi*	FJ859902	-	-
Trichoptera	Integripalpia	Phryganeoidea	Phryganeidae	*Eubasilissa regina*	KF756943	-	-
Lepidoptera	Glossata	Papilionoidea	Nymphalidae	*Abrota ganga*	KF590536	-	-
Orthoptera	Caelifera	Tetrigoidea	Tetrigidae	*Alulatettix yunnanensis*	JQ272702	-	-
Orthoptera	Caelifera	-	Acrididae	*Acrida cinerea*	GU344100	-	-
Hemiptera	Auchenorrhyncha	Cercopoidea	Cercopidae	*Callitettix biformis*	JX844627	-	-
Hemiptera	Heteroptera	Coreoidea	Rhopalidae	*Aeschyntelus notatus*	EU427333	-	-
Grylloblattodea	Grylloblattidae	-	Grylloblattidae	*Grylloblatta sculleni*	DQ241796	-	-
Phasmatodea	Anareolatae	-	Phasmatidae	*Extatosoma tiaratum*	AB642680	-	-
Mantophasmatodea	-	-	Mantophasmatidae	*Sclerophasma paresisensis*	DQ241798	-	-
Mantodea	-	-	Hymenopodidae	*Anaxarcha zhengi*	KU201320	-	-
Blattodea	-	Blaberoidea	Ectobiidae	*Blattella bisignata*	JX233805	-	-
Blattodea	Blattoidea	Blattoidea	Termitoidae	*Aciculitermes aciculatus*	KY224548	-	-

Note: Bold indicates the species newly sequenced in this study. "-" indicates no application.

Table S2. (A) The partition schemes and best-fitting models selected by PartitionFinder for dataset of 85taxa_PCG$1_{NT}2_{NT}3_{RY}$RNA.

85taxa_PCG$1_{NT}2_{NT}3_{RY}$RNA			
**Subset**	**Partition names**	**Sites**	**Model**
Partition 1	nad5, nad1, nad4l, nad4	4269	GTR+I+G
Partition 2	cob, cox3	1911	GTR+I+G
Partition 3	atp8, nad2, nad6	1659	GTR+I+G
Partition 4	nad3, atp6	1026	GTR+I+G
Partition 5	cox1	1527	GTR+I+G
Partition 6	cox2	678	GTR+I+G
Partition 7	rrna	2013	GTR+I+G
Partition 8	trna	2135	GTR+I+G

Abbreviations used in the BestModel: GTR, General-Time-Reversible model; I, invariant sites; G, discrete Gamma distribution.

Table S2. (B) The partition schemes and best-fitting models selected by PartitionFinder for dataset of 85taxa_PCG$1_{NT}2_{NT}$RNA.

85taxa_PCG$1_{NT}2_{NT}$RNA			
**Subset**	**Partition names**	**Sites**	**Model**
Partition 1	nad4, nad4l, nad1, nad5	2846	GTR+I+G
Partition 2	nad3, cob, atp6	1438	GTR+I+G
Partition 3	atp8, nad2, nad6	1106	GTR+I+G
Partition 4	cox3, cox1	1538	GTR+I+G
Partition 5	cox2	452	GTR+I+G
Partition 6	rrna	2013	GTR+I+G
Partition 7	trna	2135	GTR+I+G

Table S2. (C) The partition schemes and best-fitting models selected by PartitionFinder for dataset of 79taxa_PCG1$_{NT}$2$_{NT}$3$_{RY}$RNA.

79taxa_PCG1$_{NT}$2$_{NT}$3$_{RY}$RNA			
**Subset**	**Partition names**	**Sites**	**Model**
Partition 1	nad4l, nad4, nad1, nad5	4269	GTR+I+G
Partition 2	cob, cox3, atp6	2586	GTR+I+G
Partition 3	atp8, nad2, nad6	1659	GTR+I+G
Partition 4	nad3	351	GTR+I+G
Partition 5	cox1	1527	GTR+I+G
Partition 6	cox2	678	GTR+I+G
Partition 7	rrna	2013	GTR+I+G
Partition 8	trna	2135	GTR+I+G

Table S2. (D) The partition schemes and best-fitting models selected by PartitionFinder for dataset of 79taxa_PCG1$_{NT}$2$_{NT}$RNA.

79taxa_PCG1$_{NT}$2$_{NT}$RNA			
**Subset**	**Partition names**	**Sites**	**Model**
Partition 1	nad4, nad4l, nad1, nad5	2846	GTR+I+G
Partition 2	nad3, cob, atp6	1438	GTR+I+G
Partition 3	atp8, nad2, nad6	1106	GTR+I+G
Partition 4	cox3, cox1	1538	GTR+I+G
Partition 5	cox2	452	GTR+I+G
Partition 6	rrna	2013	GTR+I+G
Partition 7	trna	2135	GTR+I+G

Table S3. Summary of the phylogenetic studies on the Palaeoptera problem.

References	Hypothesis supported	Samples included			Data type	Phylogenetic reconstruction method	Model used	Ref. number in this study
		Odo	Eph	Neo				
Zhang et al. 2008	Eph + (Odo + Neo)	1	1	7	mitogenome data	Maximum parsimony analyses of nucleotide and protein sequences of 12 mitochondrial PCGs (PAUP), ML analyses of protein sequences of 12 mitochondrial PCGs (PhyML), Bayesian analyses of nucleotide and protein sequences of 12 mitochondrial PCGs (MrBayes).	MtArt+I+G (ML), GTR+I+G for nucleotide and MtRev+I+G for protein (BI)	36
Comandi et al. 2009	Odo + Neo	1	0	6		Bayesian inference (MrBayes) of protein sequences of 13 PCGs.	MtPan+I	28
Lin et al. 2010	Odo + (Neo including Eph)	3	2	8		Maximum parsimony analyses of nucleotide and protein sequences of 13 PCGs (PAUP), Maximum likelihood analyses of nucleotide (RAxML) and protein (PhyML) sequences of 13 PCGs, Bayesian analyses of nucleotide and protein sequences of 13 PCGs (MrBayes).	GTRCAT+I+G (ML-nucleotide), MtArt+I+G (ML-protein), complex model (BI-nucleotide), MtRev+G+F (BI-protein)	31
Zhang et al. 2010	Odo + (Neo including Eph)	1	1	10		Bayesian inference using protein sequences of 13 PCGs (MrBayes).	-	79

Li et al. 2014	Odo + (Eph + Neo)	3	4	16		Bayesian (MrBayes) and maximum likelihood (RAxML) analyses based on nucleotide sequences of 13 PCGs (1st codon and 2nd codon positions).	GTR+I+G for both BI and ML analyses	70
Rutschmann et al. 2017	Eph + (Odo + Neo) or (Odo + Eph) + Neo	10	19	51		Bayesian (MrBayes) and maximum likelihood (RAxML) analyses based on protein sequences of 13 PCGs.	MTZOA+G+I and MTZOA+G+I+F both BI and ML analyses	68
Cai et al. 2018	Eph + (Odo + Neo)	27	20	16		Bayesian (MrBayes) and maximum likelihood (PhyML) Inferences using nucleotide sequences of 13 PCGs.	-	27
Present study	(Odo + Eph) + Neo	31	18	20		Maximum likelihood (IQ-TREE) and Bayesian analyses (PhyloBayes) based on the nucleotide sequences of 37 mitochondrial genes.	GTR for ML, CAT-GTR for BI analyses	-
Misof et al. 2007	Odo + (Eph + Neo)	44	16	230	18S rRNA	Bayesian analyses (PHASE) based on 18S rRNA sequences.	complex RNA models	12
Ishiwata et al. 2010	(Odo + Eph) + Neo	2	2	38	three nuclear protein-coding genes	ML analyses (RAxML) and Bayesian inferences (MrBayes) based on three nuclear protein-coding gene sequences.	WAGCAT+F and WAG+G+F (ML), WAG+G (BI)	7
Simon et al. 2009	Odo + (Eph + Neo)	1	1	12	EST	ML analyses (RAxML) and Bayesian inferences (MrBayes) based on data from expressed sequence tag (125 genes, and 31,643 amino acid positions).	WAG (ML), WAG+I+G (BI)	26

Regier et al. 2010	(Odo + Eph) + Neo	2	2	6		Bayesian (MrBayes), maximum likelihood (GARLI) and parsimony (PAUP) analyses based on nucleotide and protein sequences from 62 single-copy nuclear protein-coding genes.	GTR+I+G for nucleotide and JTT+G for protein	8
					genome-scale data			
Misof et al. 2014	(Odo + Eph) + Neo	3	4	114		ML analyses (ExaML) based on nucleotide and amino acid sequences from 1478 single-copy nuclear genes generated from genomes and transcriptomes.	nucleotide applying a site-specific rate model, and amino acid applying a protein domain–based partitioning scheme to improve the biological realism of the applied evolutionary models	10
Blanke et al. 2012	(Odo + Eph) + Neo	8	4	19	morphological data	Bayesian (MrBayes), maximum likelihood (RAxML) and parsimony (TNT) analyses based on head characters.	Mk for BI analyses, Mkv for ML analyses	16

Kjer et al. 2006	(Odo + Eph) + Neo	7	7	101	combining data of molecular and morphological characters	Bayesian (MrBayes) and parsimony (PAUP) analyses based on combined data including a fragment of the 28S (D1–D8) and complete sequences for the 18S, histone (H3), EF-1α, COI, COII, the 12S and 16S plus the intervening tRNA, and 170 morphological characters.	GTR+I+G model for nucleotides, and MK model for the morphology	9

Note: Odo, Odonata; Eph, Ephemeroptera; Neo, Neoptera. "-" indicates that the model is not presented in the reference, or the item is not applied.

Figure legends for supplementary figures

Fig. S1 Phylogenetic tree based on maximum likelihood bootstrap analysis of the nucleotide sequence dataset of 85taxa_PCG$1_{NT}2_{NT}3_{RY}$RNA, under the GTR model. Values at nodes are bootstrap scores (Left: the values from dataset of 85taxa_PCG$1_{NT}2_{NT}3_{RY}$RNA, right: the values from dataset of 85taxa_PCG$1_{NT}2_{NT}$RNA). "--" indicates the node not being recovered by the dataset of 85taxa_PCG$1_{NT}2_{NT}$RNA. Scale bar represents substitutions/site. The meaning of color is as Fig. 2.

Fig. S2 Phylogenetic tree based on maximum likelihood bootstrap analysis of the nucleotide sequence dataset of 79taxa_PCG$1_{NT}2_{NT}3_{RY}$RNA, under the GTR model. Values at nodes are bootstrap scores (Left: the values from dataset of 79taxa_PCG$1_{NT}2_{NT}3_{RY}$RNA, right: the values from dataset of 79taxa_PCG$1_{NT}2_{NT}$RNA). "--" indicates the node not being recovered by the dataset of 79taxa_PCG$1_{NT}2_{NT}$RNA. Scale bar represents substitutions/site. The meaning of color is as Fig. 2.

Fig. S3 Phylogenetic tree based on Bayesian inference of the nucleotide sequence dataset of 79taxa_PCG$1_{NT}2_{NT}3_{RY}$RNA, under the CAT+GTR model. Values at nodes are Bayesian posterior probability support (Left: the values from dataset of 79taxa_PCG$1_{NT}2_{NT}3_{RY}$RNA, right: the values from dataset of 79taxa_PCG$1_{NT}2_{NT}$RNA). "--" indicates the node not being recovered by the dataset of 79taxa_PCG$1_{NT}2_{NT}$RNA. Scale bar represents substitutions/site. The meaning of color is as Fig. 2.

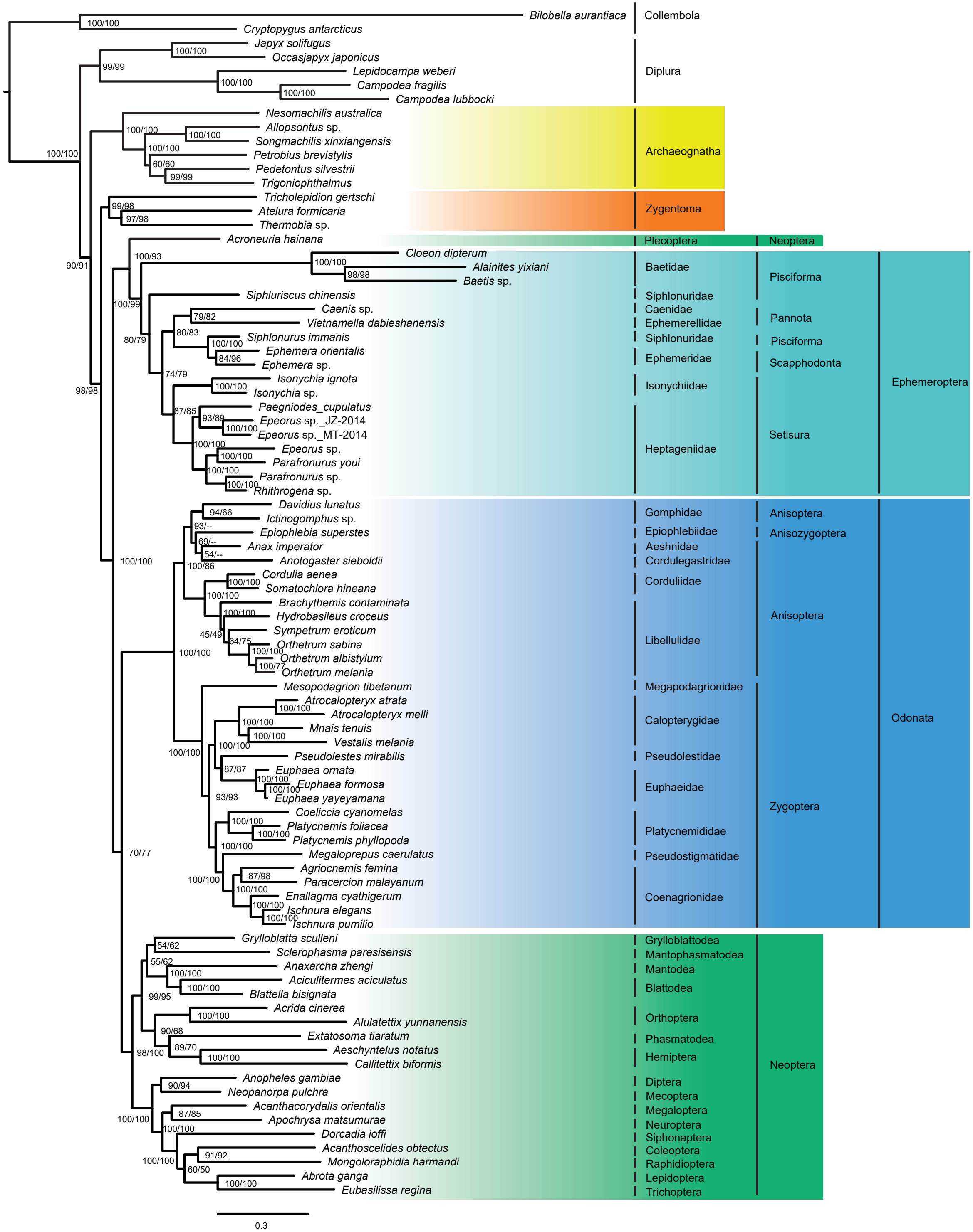
Bilobella aurantiaca
Cryptopygus antarcticus
Collembola
Japyx solifugus
Occasjapyx japonicus
Lepidocampa weberi
Campodea fragilis
Campodea lubbocki
Diplura
Nesomachilis australica
Allopsontus sp.
Songmachilis xinxiangensis
Petrobius brevistylis
Pedetontus silvestrii
Trigoniophthalmus
Archaeognatha
Tricholepidion gertschi
Atelura formicaria
Thermobia sp.
Zygentoma
Acroneuria hainana
Plecoptera
Neoptera
Cloeon dipterum
Alainites yixiani
Baetis sp.
Baetidae
Pisciforma
Siphluriscus chinensis
Siphlonuridae
Caenis sp.
Caenidae
Vietnamella dabieshanensis
Ephemerellidae
Pannota
Siphlonurus immanis
Siphlonuridae
Pisciforma
Ephemera orientalis
Ephemera sp.
Ephemeridae
Scapphodonta
Isonychia ignota
Isonychia sp.
Isonychiidae
Paegniodes_cupulatus
Epeorus sp._JZ-2014
Epeorus sp._MT-2014
Epeorus sp.
Parafronurus youi
Parafronurus sp.
Rhithrogena sp.
Heptageniidae
Setisura
Ephemeroptera
Davidius lunatus
Ictinogomphus sp.
Gomphidae
Anisoptera
Epiophlebia superstes
Epiophlebiidae
Anisozygoptera
Anax imperator
Aeshnidae
Anotogaster sieboldii
Cordulegastridae
Cordulia aenea
Somatochlora hineana
Corduliidae
Brachythemis contaminata
Hydrobasileus croceus
Sympetrum eroticum
Orthetrum sabina
Orthetrum albistylum
Orthetrum melania
Libellulidae
Anisoptera
Mesopodagrion tibetanum
Megapodagrionidae
Atrocalopteryx atrata
Atrocalopteryx melli
Mnais tenuis
Vestalis melania
Calopterygidae
Pseudolestes mirabilis
Pseudolestidae
Euphaea ornata
Euphaea formosa
Euphaea yayeyamana
Euphaeidae
Coeliccia cyanomelas
Platycnemis foliacea
Platycnemis phyllopoda
Platycnemididae
Megaloprepus caerulatus
Pseudostigmatidae
Agriocnemis femina
Paracercion malayanum
Enallagma cyathigerum
Ischnura elegans
Ischnura pumilio
Coenagrionidae
Zygoptera
Odonata
Grylloblatta sculleni
Grylloblattodea
Sclerophasma paresisensis
Mantophasmatodea
Anaxarcha zhengi
Mantodea
Aciculitermes aciculatus
Blattella bisignata
Blattodea
Acrida cinerea
Alulatettix yunnanensis
Orthoptera
Extatosoma tiaratum
Phasmatodea
Aeschyntelus notatus
Callitettix biformis
Hemiptera
Anopheles gambiae
Diptera
Neopanorpa pulchra
Mecoptera
Acanthacorydalis orientalis
Megaloptera
Apochrysa matsumurae
Neuroptera
Dorcadia ioffi
Siphonaptera
Acanthoscelides obtectus
Coleoptera
Mongoloraphidia harmandi
Raphidioptera
Abrota ganga
Lepidoptera
Eubasilissa regina
Trichoptera
Neoptera
100/100
100/100
99/99
100/100
100/100
100/100
100/100
100/100
100/100
60/60
99/99
90/91
99/98
97/98
100/93
100/100
98/98
100/99
79/82
80/83
80/79
100/100
84/96
74/79
98/98
100/100
87/85
93/89
100/100
100/100
100/100
100/100
100/100
94/66
93/--
69/--
54/--
100/86
100/100
100/100
100/100
100/100
45/49
64/75
100/100
100/77
100/100
100/100
100/100
100/100
100/100
100/100
87/87
100/100
100/100
93/93
100/100
100/100
100/100
70/77
87/98
100/100
100/100
100/100
100/100
54/62
55/62
100/100
100/100
99/95
100/100
90/68
89/70
98/100
100/100
90/94
87/85
100/100
100/100
91/92
100/100
60/50
100/100
0.3

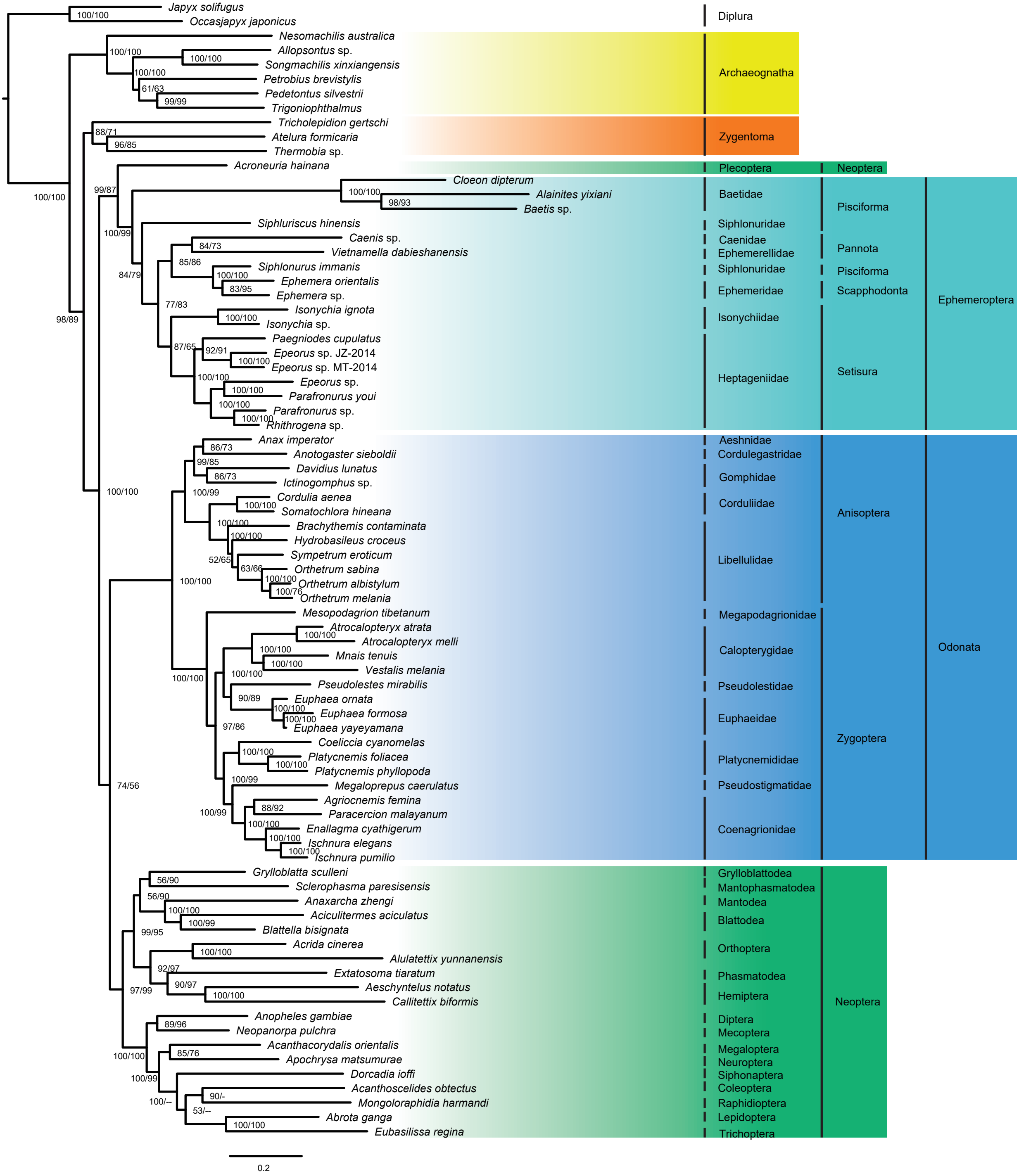

Japyx solifugus
Occasjapyx japonicus
Diplura
Nesomachilis australica
Allopsontus sp.
Songmachilis xinxiangensis
Petrobius brevistylis
Pedetontus silvestrii
Trigoniophthalmus
Archaeognatha
Tricholepidion gertschi
Atelura formicaria
Thermobia sp.
Zygentoma
Acroneuria hainana
Plecoptera
Neoptera
Cloeon dipterum
Alainites yixiani
Baetis sp.
Baetidae
Pisciforma
Siphluriscus hinensis
Siphlonuridae
Caenis sp.
Vietnamella dabieshanensis
Caenidae
Ephemerellidae
Pannota
Siphlonurus immanis
Ephemera orientalis
Ephemera sp.
Siphlonuridae
Ephemeridae
Pisciforma
Scapphodonta
Ephemeroptera
Isonychia ignota
Isonychia sp.
Isonychiidae
Paegniodes cupulatus
Epeorus sp. JZ-2014
Epeorus sp. MT-2014
Epeorus sp.
Parafronurus youi
Parafronurus sp.
Rhithrogena sp.
Heptageniidae
Setisura
Anax imperator
Anotogaster sieboldii
Davidius lunatus
Ictinogomphus sp.
Aeshnidae
Cordulegastridae
Gomphidae
Cordulia aenea
Somatochlora hineana
Corduliidae
Brachythemis contaminata
Hydrobasileus croceus
Sympetrum eroticum
Orthetrum sabina
Orthetrum albistylum
Orthetrum melania
Libellulidae
Anisoptera
Mesopodagrion tibetanum
Megapodagrionidae
Atrocalopteryx atrata
Atrocalopteryx melli
Mnais tenuis
Vestalis melania
Calopterygidae
Pseudolestes mirabilis
Pseudolestidae
Euphaea ornata
Euphaea formosa
Euphaea yayeyamana
Euphaeidae
Coeliccia cyanomelas
Platycnemis foliacea
Platycnemis phyllopoda
Platycnemididae
Megaloprepus caerulatus
Pseudostigmatidae
Agriocnemis femina
Paracercion malayanum
Enallagma cyathigerum
Ischnura elegans
Ischnura pumilio
Coenagrionidae
Zygoptera
Odonata
Grylloblatta sculleni
Sclerophasma paresisensis
Anaxarcha zhengi
Aciculitermes aciculatus
Blattella bisignata
Grylloblattodea
Mantophasmatodea
Mantodea
Blattodea
Acrida cinerea
Alulatettix yunnanensis
Orthoptera
Extatosoma tiaratum
Aeschyntelus notatus
Callitettix biformis
Phasmatodea
Hemiptera
Anopheles gambiae
Neopanorpa pulchra
Diptera
Mecoptera
Acanthacorydalis orientalis
Apochrysa matsumurae
Megaloptera
Neuroptera
Dorcadia ioffi
Acanthoscelides obtectus
Mongoloraphidia harmandi
Abrota ganga
Eubasilissa regina
Siphonaptera
Coleoptera
Raphidioptera
Lepidoptera
Trichoptera
Neoptera
100/100
98/89
88/71
96/85
61/63
99/99
99/87
100/99
84/79
85/86
84/73
83/95
77/83
87/65
92/91
100/100
98/93
86/73
99/85
100/99
52/65
63/66
100/76
74/56
90/89
97/86
100/99
88/92
56/90
99/95
92/97
90/97
97/99
89/96
85/76
100/99
100/--
90/-
53/--
0.2

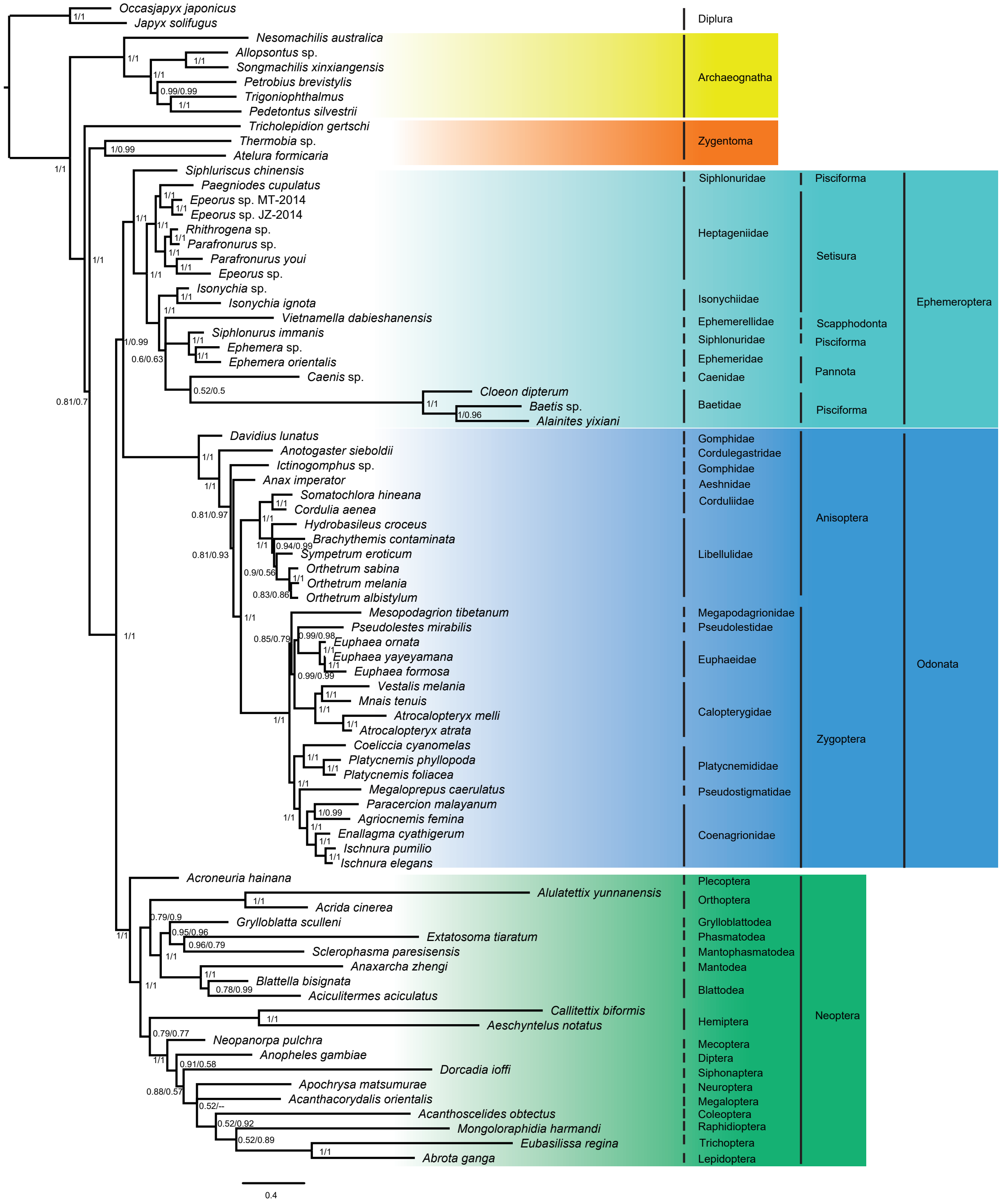
Occasjapyx japonicus
Japyx solifugus
Nesomachilis australica
Allopsontus sp.
Songmachilis xinxiangensis
Petrobius brevistylis
Trigoniophthalmus
Pedetontus silvestrii
Tricholepidion gertschi
Thermobia sp.
Atelura formicaria
Siphluriscus chinensis
Paegniodes cupulatus
Epeorus sp. MT-2014
Epeorus sp. JZ-2014
Rhithrogena sp.
Parafronurus sp.
Parafronurus youi
Epeorus sp.
Isonychia sp.
Isonychia ignota
Vietnamella dabieshanensis
Siphlonurus immanis
Ephemera sp.
Ephemera orientalis
Caenis sp.
Cloeon dipterum
Baetis sp.
Alainites yixiani
Davidius lunatus
Anotogaster sieboldii
Ictinogomphus sp.
Anax imperator
Somatochlora hineana
Cordulia aenea
Hydrobasileus croceus
Brachythemis contaminata
Sympetrum eroticum
Orthetrum sabina
Orthetrum melania
Orthetrum albistylum
Mesopodagrion tibetanum
Pseudolestes mirabilis
Euphaea ornata
Euphaea yayeyamana
Euphaea formosa
Vestalis melania
Mnais tenuis
Atrocalopteryx melli
Atrocalopteryx atrata
Coeliccia cyanomelas
Platycnemis phyllopoda
Platycnemis foliacea
Megaloprepus caerulatus
Paracercion malayanum
Agriocnemis femina
Enallagma cyathigerum
Ischnura pumilio
Ischnura elegans
Acroneuria hainana
Alulatettix yunnanensis
Acrida cinerea
Grylloblatta sculleni
Extatosoma tiaratum
Sclerophasma paresisensis
Anaxarcha zhengi
Blattella bisignata
Aciculitermes aciculatus
Callitettix biformis
Aeschyntelus notatus
Neopanorpa pulchra
Anopheles gambiae
Dorcadia ioffi
Apochrysa matsumurae
Acanthacorydalis orientalis
Acanthoscelides obtectus
Mongoloraphidia harmandi
Eubasilissa regina
Abrota ganga
Diplura
Archaeognatha
Zygentoma
Siphlonuridae
Heptageniidae
Isonychiidae
Ephemerellidae
Siphlonuridae
Ephemeridae
Caenidae
Baetidae
Pisciforma
Setisura
Scapphodonta
Pisciforma
Pannota
Pisciforma
Ephemeroptera
Gomphidae
Cordulegastridae
Gomphidae
Aeshnidae
Corduliidae
Libellulidae
Megapodagrionidae
Pseudolestidae
Euphaeidae
Calopterygidae
Platycnemididae
Pseudostigmatidae
Coenagrionidae
Anisoptera
Zygoptera
Odonata
Plecoptera
Orthoptera
Grylloblattodea
Phasmatodea
Mantophasmatodea
Mantodea
Blattodea
Hemiptera
Mecoptera
Diptera
Siphonaptera
Neuroptera
Megaloptera
Coleoptera
Raphidioptera
Trichoptera
Lepidoptera
Neoptera
0.4
